# Office-Based Addiction Treatment Retention and Mortality Among People Experiencing Homelessness

**DOI:** 10.1001/jamanetworkopen.2021.0477

**Published:** 2021-03-04

**Authors:** Danielle R. Fine, Elizabeth Lewis, Karen Weinstock, Joseph Wright, Jessie M. Gaeta, Travis P. Baggett

**Affiliations:** 1 Division of General Internal Medicine, Department of Medicine, Massachusetts General Hospital, Boston; 2 Harvard Medical School, Boston, Massachusetts; 3 Institute for Research, Quality, and Policy in Homeless Health Care, Boston Health Care for the Homeless Program, Boston, Massachusetts; 4 Boston University School of Public Health, Boston, Massachusetts; 5 Section of General Internal Medicine, Department of Medicine, Boston University School of Medicine, Boston, Massachusetts

## Abstract

**Importance:**

People experiencing homelessness have been disproportionately affected by the opioid overdose crisis. To mitigate morbidity and mortality, several office-based addiction treatment (OBAT) programs designed for this population have been established across the US, but studies have not yet evaluated their outcomes.

**Objective:**

To evaluate treatment retention and mortality in an OBAT program designed specifically for individuals experiencing homelessness with opioid use disorder (OUD).

**Design, Setting, and Participants:**

A retrospective cohort study was conducted in the Boston Health Care for the Homeless Program (BHCHP). Participants included all adult patients (N = 1467) who had 1 or more OBAT program encounter at BHCHP from January 1 through December 31, 2018. Data analysis was conducted from January 13 to December 14, 2020.

**Exposures:**

Sociodemographic, clinical, and addiction treatment–related characteristics were abstracted from the BHCHP electronic health record.

**Main Outcomes and Measures:**

The primary outcome was all-cause mortality, identified by linkage to the Massachusetts Department of Public Health vital records. Multivariable Cox proportional hazards regression analyses were performed to evaluate baseline and time-varying variables associated with all-cause mortality. Secondary addiction treatment–related outcomes were abstracted from the electronic health record and included (1) BHCHP OBAT program retention, (2) buprenorphine continuation and adherence verified by toxicology testing, and (3) opioid abstinence verified by toxicology testing.

**Results:**

Of 1467 patients in the cohort, 1046 were men (71.3%) and 731 (49.8%) were non-Hispanic White; mean (SD) age was 42.2 (10.6) years. Continuous retention in the OBAT program was 45.2% at 1 month, 21.7% at 6 months, and 11.3% at 12 months. Continuous buprenorphine adherence was 41.5% at 1 month, 17.6% at 6 months, and 10.2% at 12 months, and continuous opioid abstinence was 28.3% at 1 month, 6.1% at 6 months, and 2.9% at 12 months. The all-cause mortality rate was 29.0 deaths per 1000 person-years, with 51.8% dying from drug overdose. Past-month OBAT program attendance was associated with lower mortality risk (adjusted hazard ratio, 0.34; 95% CI, 0.21-0.55).

**Conclusions and Relevance:**

Mortality rates were high in this cohort of addiction treatment–seeking homeless and unstably housed individuals with OUD. Although continuous OBAT program retention was low, past-month attendance in care was associated with reduced mortality risk. Future work should examine interventions to promote increased OBAT attendance to mitigate morbidity and mortality in this vulnerable population.

## Key Points

**Question:**

What are the retention rates in an office-based addiction treatment program designed specifically for people experiencing homelessness, and is program attendance associated with reduced mortality risk?

**Findings:**

In this cohort study of 1467 homeless or unstably housed adults with opioid use disorder, retention in the office-based addiction treatment program was 45.2% at 1 month, 21.7% at 6 months, and 11.3% at 12 months. Past-month office-based addiction treatment attendance was associated with a 66% reduction in mortality risk.

**Meaning:**

Interventions to promote increased attendance in addiction treatment among people experiencing homelessness are needed to mitigate morbidity and mortality in this population.

## Teaser

This cohort study examines the association between mortality and retention within office-based addiction treatment settings in individuals with opioid use disorder who are experiencing homelessness.

## Introduction

People experiencing homelessness have been disproportionately affected by the opioid overdose crisis, experiencing overdose rates up to 30-fold higher than the general population.^1,2,3,4^ This disparity can be attributed to increased rates of opioid use disorder (OUD) and barriers to accessing addiction treatment.^5^

Medications for OUD, specifically methadone and buprenorphine, have become the mainstay of treatment for patients with OUD, including homeless and marginally housed individuals.^5^ However, there are several barriers that prevent homeless individuals from receiving this evidence-based treatment, including fragmented and stigmatizing medical care, especially around episodes of incarceration, and the unfounded belief that treatment is unsuccessful in people who lack stable housing.^6,7,8,9,10^

To overcome these barriers, innovative methods, such as provision of medications for OUD within harm reduction agencies, mobile methadone vans, and buprenorphine prescribing by street medicine teams, have been deployed and demonstrate the ability to reach larger populations of homeless and unstably housed patients.^9,11,12,13^ Because brick-and-mortar clinics may be more sustainable, there has been increased interest in developing office-based addiction treatment (OBAT) programs specifically for people experiencing homelessness.^5^ Several OBAT programs for this population have been established across the US, but to our knowledge, studies have not yet evaluated their outcomes or effectiveness. Given the potential for such programs to mitigate morbidity and mortality in this high-risk group of individuals, rigorous evaluations of their performance are required.

We aimed to perform one of the first evaluations of treatment outcomes and mortality in an OBAT program designed specifically for people experiencing homelessness. We hypothesized that OBAT program attendance would be associated with reduced mortality risk. Furthermore, we aimed to identify points in care that were particularly vulnerable to patient dropout and identify characteristics associated with treatment retention.

## Methods

### Study Design and Population

We conducted a retrospective cohort study of all adults (age ≥18 years) who had at least 1 OBAT program encounter at the Boston Health Care for the Homeless Program (BHCHP) between January 1 and December 31, 2018. The BHCHP cares for more than 11 000 patients annually at more than 40 locations in the greater Boston, Massachusetts, area.^14^ Patients must be homeless to enroll in care at BHCHP but may elect to continue receiving care there after they are no longer homeless.

The BHCHP OBAT program was designed for patients with OUD and was fashioned after the Massachusetts model, with nurse care managers playing a central role.^15^ The program was launched in 2006 at BHCHP’s largest outpatient clinic site and, in response to increased fentanyl-related overdoses, expanded in 2013 to deliver lower-threshold care at several shelter sites throughout the city of Boston.^16^ Buprenorphine is usually prescribed at the first visit unless a nurse intake visit precedes the initial prescriber evaluation, which occurred more frequently during the earlier years of the program. Subsequent medical visits are typically staffed by nurse care managers who manage refills, obtain samples for toxicologic testing, and discuss the need for dose adjustments with prescribers. Behavioral health visits, which are not required, are primarily staffed by psychotherapists integrated within the OBAT team.

The cohort entry date was the date of the first OBAT encounter, and the follow-up window extended through January 31, 2019, to allow for at least 1 full year of follow-up after the last possible entry date. Patients were followed up until the primary outcome (death) or the end of the study period. Data were deidentified, and analysis was conducted from January 13 to December 14, 2020. The Partners HealthCare Institutional Review Board approved this study with a waiver of informed consent. Consent was waived because the study did not involve more than minimal risk to participants, would not adversely affect the rights and welfare of the participants, and could not be carried out practicably without this waiver. This study followed the Strengthening the Reporting of Observational Studies in Epidemiology (STROBE) reporting guideline for cohort studies.^17^

### Outcomes

The primary outcome was all-cause mortality, identified by cross-linking the BHCHP cohort with the Massachusetts Department of Public Health’s Registry of Vital Records and Statistics. We used LinkPlus, version 2.0, a probabilistic record linkage program that computes linkage probability scores for possible record pairs based on the level of agreement and relative importance of various personal identifiers.^18^ Two investigators (D.R.F. and K.W.) each manually reviewed record pairs that achieved a probability score of 7 or higher^18^ and classified a pair as a true linkage if it matched on 1 of the following National Death Index criteria: (1) social security number, (2) first and last name, with month and year of birth (±1 year), or (3) first and last name with month and day of birth.^19^ The 2 investigators achieved perfect concordance and interrater reliability (κ = 1.00).

We ascertained cause of death based on *International Statistical Classification of Diseases and Related Health Problems, Tenth Revision* (*ICD-10*) underlying cause of death codes (eTable 1 in the Supplement). We were not able to obtain cause of death information for deaths that occurred in 2019 owing to a legislative rule prohibiting their release for 3 years. We defined drug overdose as drug poisoning deaths that were unintentional (*ICD-10* codes X40-X44) or of undetermined intent (*ICD-10* codes Y10-Y14), as in earlier studies.^1,20^ We examined the multiple cause of death fields to determine which substances were implicated in each drug overdose.

### Addiction Treatment–Related Outcomes

We used the cascade of care model to evaluate addiction treatment–related secondary outcomes. The cascade of care model in addiction medicine outlines key steps in the treatment and recovery process to facilitate the identification of points that are the most vulnerable to dropouts in care.^21,22^ Cascade of care outcomes included each of the following, abstracted from the BHCHP electronic health record using automated methods: (1) OBAT program retention (based on program attendance via in-person visits with a BHCHP OBAT physician, nurse care manager, or behavioral health care professional independent of buprenorphine prescriptions); (2) buprenorphine initiation (based on the first buprenorphine prescription during the study period), continuation (defined as having subsequent buprenorphine prescriptions), and adherence (defined as having a positive buprenorphine urine toxicologic screen); and (3) opioid abstinence (defined as having no detectable opioids on urine toxicologic testing). Several of these treatment-related outcomes also served as independent variables in the analysis of the primary outcome of mortality.

### Covariates

Covariates were selected a priori and abstracted from the electronic health record using automated methods. Sociodemographic characteristics included age, sex, race/ethnicity, insurance status, and housing status as recorded in the baseline clinical encounter, dichotomized into literal homelessness (shelter or street) vs other living situations. Clinical characteristics included comorbid conditions, medications, and addiction treatment–related characteristics. Comorbid conditions were defined as having an *International Classification of Diseases, Ninth Revision* or *ICD-10* code from validated *ICD* code algorithms within 12 months before cohort entry (eTable 2 in the Supplement). These codes included a Charlson comorbidity index score, a previous serious bacterial infection (a composite of common injection drug use–related infections, including endocarditis, osteomyelitis, septic arthritis, and epidural abscess), cold-related injury,^23^ alcohol use disorder, other drug use disorder, and serious mental illness (schizophrenia or bipolar disorder). Medication prescriptions included benzodiazepines, opioids (excluding buprenorphine), antidepressants, antipsychotics, and sedatives, all treated as time varying and measured on a monthly basis. Baseline addiction treatment–related characteristics included site of the first OBAT encounter (clinic vs shelter/outreach site) and year of the first OBAT encounter, dichotomized into 2008-2013 vs 2014-2018 based on secular changes in opioid overdose rates in Boston and a corresponding shift toward a low-barrier model of care (ie, flexible attendance and urine drug testing requirements to prioritize a reduction in drug-related harms over abstinence). Time-varying addiction treatment–related characteristics included OBAT program attendance, buprenorphine adherence, and opioid abstinence, as previously defined and measured on a monthly basis.

### Statistical Analysis

We used descriptive statistics to present patient characteristics. We calculated all-cause mortality rates and tabulated the leading causes of death. We then performed multivariable Cox proportional hazards regression analyses to identify baseline and time-varying characteristics associated with mortality, reporting hazard ratios (HRs) and 95% CIs. We tested the proportional hazards assumption by evaluating Schoenfeld residuals.

We included covariates in our model based on a priori clinical knowledge. We excluded variables that were highly collinear and variables with a limited number of outcomes because we would not be able to establish associations based on those results. Covariates that had a high degree of collinearity were OBAT program attendance, buprenorphine adherence, and opioid abstinence. This degree of collinearity was due in part to missingness of buprenorphine adherence and opioid abstinence data, which are likely not missing at random but missing largely owing to a lack of follow-up. We therefore included only OBAT program attendance in the primary model; a sensitivity analysis included all 3 treatment-related covariates using a missing indicator method for missing data. To account for patients who had multiple OBAT encounters during each month, we also performed a sensitivity analysis using the number of OBAT encounters as a continuous variable.

Next, we used descriptive statistics to assess addiction treatment–related outcomes at 1, 3, 6, 9, and 12 months. We report continuous frequencies of these outcomes. We also calculated the sum and percentage of positive buprenorphine toxicologic tests and the sum and percentage of negative opioid toxicologic tests for each individual. In addition, we performed multivariable logistic regression to identify baseline characteristics independently associated with OBAT retention at 1 month, reporting odds ratios (ORs) and 95% CIs. A 2-sided threshold of *P* < .05 was considered statistically significant. All analyses were performed using RStudio, version 1.2.5033 (2009-2019 RStudio, PBC).

## Results

### Baseline Cohort Characteristics

Among the 1467 adults in the cohort, the mean (SD) age at study entry was 42.2 (10.6) years, 1046 were men (71.3%), 421 were women (28.7%), 731 (49.8%) were non-Hispanic White, 442 (30.1%) were Hispanic, and 183 (12.5%) were non-Hispanic Black. Most participants (1266 [86.3%]) had public insurance, and approximately half (719 [49.0%]) were homeless at baseline. A total of 275 participants (18.8%) had a diagnosis of alcohol use disorder, 1202 (81.9%) had a diagnosis of another drug use disorder, and 189 (12.9%) had a diagnosis of serious mental illness. Most patients had their first OBAT encounter at a clinic-based site (1181 [80.5%]) between 2014 and 2018 (1018 [69.4%]) (Table 1).

**Table 1.** Characteristics of People Experiencing Homelessness Who Engaged in an OBAT Program, 2008-2018

Patient characteristic	All patients (N = 1467)
**Sociodemographic characteristic**	
Age, mean (SD)	42.2 (10.6)
Sex, No. (%)	
Male	1046 (71.3)
Female	421 (28.7)
Race/ethnicity, No. (%)	
Non-Hispanic White	731 (49.8)
Non-Hispanic Black	183 (12.5)
Hispanic	442 (30.1)
Other/unknown	111 (7.6)
Insurance type, No. (%)	
Private	15 (1.0)
Public	1266 (86.3)
Dual	130 (8.9)
Uninsured	56 (3.8)
Housing status, No. (%)	
Shelter/street	719 (49.0)
Other^a^	748 (51.0)
**Clinical characteristics**	
Charlson comorbidity index, median (IQR)	0 (0-1)
Serious bacterial infection, No. (%)^b^	19 (1.3)
Other substance use disorders, No. (%)	
Alcohol use disorder	275 (18.8)
Other drug use disorder	1202 (81.9)
Serious mental illness, No. (%)^c^	189 (12.9)
Cold-related injury, No. (%)^d^	31 (2.1)
Medication prescriptions, No. (%)	
Benzodiazepine	25 (1.7)
Opioid^e^	17 (1.2)
Antidepressant	71 (4.8)
Antipsychotic	37 (2.5)
Other sedating medications^f^	23 (1.6)
Naloxone	7 (0.5)
**Addiction treatment–related characteristics**	
Year of first OBAT encounter, No. (%)	
2008-2013	449 (30.6)
2014-018	1018 (69.4)
Site of first OBAT encounter, No. (%)	
Clinic	1181 (80.5)
Shelter/outreach	286 (19.5)
No. of OBAT encounters, median (IQR)	4 (0-17)
Positive buprenorphine toxicologic tests, median (IQR), %	100 (80-100)
Negative opioid toxicologic tests, median (IQR), %	61 (20-100)

Abbreviations: IQR, interquartile range; OBAT, office-based addiction treatment.

^a^ Includes assisted living facility, doubled up (ie, sharing the housing of other persons owing to a lack of personal housing), housing with and without supportive services, motel, respite home, residential treatment center, skilled nursing facility, transitional housing, other housing status, and unknown housing status.

^b^ Includes endocarditis, epidural abscess, osteomyelitis, and septic arthritis.

^c^ Includes a diagnosis of bipolar disorder and/or schizophrenia.

^d^ Includes frostbite, hypothermia, and immersion foot.

^e^ Excludes buprenorphine.

^f^ Includes nonbenzodiazepine sedative-hypnotics and trazodone.

### Mortality-Related Outcomes

A total of 193 individuals (13.2%) died, with an all-cause mortality rate of 29.0 per 1000 person-years. The mortality rate remained relatively stable over the study period (eFigure 1 in the Supplement). Among deaths for which the underlying cause was available (n = 168), the leading cause was drug overdose, accounting for 87 (51.8%) of these deaths. Opioids were present in 100% of the drug overdose deaths (Table 2).

**Table 2.** Cause of Death Among 168 People Experiencing Homelessness Who Engaged in an OBAT Program^a^

Underlying cause of death	No. (%) of patients
**External causes**	
Drug overdose	87 (51.8)
Opioid	87 (100)
Alcohol	32 (36.8)
Benzodiazepine	17 (19.5)
Cocaine	15 (17.2)
Suicide	4 (2.4)
Accidents (unintentional injuries)	3 (1.8)
Homicide	2 (1.2)
Alcohol poisoning	1 (0.6)
Other accidents (nonpoisoning)	1 (0.6)
**Natural causes**	
Heart disease	15 (8.9)
Psychoactive substance use disorder	12 (7.1)
HIV disease	10 (6.0)
Cancer	6 (3.6)
Liver disease	6 (3.6)
Ill-defined conditions	5 (3.0)
Cerebrovascular disease	3 (1.8)
Anoxic brain injury	2 (1.2)
Sepsis	2 (1.2)
Viral hepatitis	2 (1.2)
Other diseases of the nervous system	2 (1.2)
Chronic lower respiratory diseases	1 (0.6)
Congenital malformations of the heart	1 (0.6)
Diabetes	1 (0.6)
Other diseases of the respiratory system	1 (0.6)
Nephritis, nephrotic syndrome, and nephrosis	1 (0.6)

Abbreviation: OBAT, office-based addiction treatment program.

^a^ Cause of death information not available for 25 deaths that occurred in 2019.

Age (adjusted HR [aHR], 1.34 per 10-year increment; 95% CI, 1.16-1.54) and a Charlson comorbidity index score of 2 or higher (aHR, 1.55; 95% CI, 1.10-2.18) were independently associated with increased hazard of all-cause mortality. Literal homelessness at baseline (aHR, 0.73; 95% CI, 0.54-0.98), having a first OBAT encounter at a shelter or outreach site (aHR, 0.58; 95% CI, 0.36-0.93), and past-month OBAT program attendance (aHR, 0.34; 95% CI, 0.21-0.55) were independently associated with a decreased hazard of all-cause mortality (Table 3). All variables met the proportional hazards assumption with the exception of sex, serious mental illness, and other drug use disorder. On removal of these variables from the model, all results remained consistent, and the global Schoenfeld test demonstrated that the proportional hazards assumption was met.

**Table 3.** Characteristics Associated With All-Cause Mortality in People Experiencing Homelessness Who Engaged in an OBAT Program

Patient characteristic	Adjusted hazard ratio (95% CI)	*P* value
Sociodemographic characteristics		
Age (per 10-y increment)	1.34 (1.16-1.54)	<.001
Sex		
Female	1 [Reference]	[Reference]
Male	0.99 (0.71-1.38)	.96
Race/ethnicity		
White	1 [Reference]	[Reference]
Non-Hispanic Black	0.67 (0.43-1.06)	.08
Hispanic	0.73 (0.52-1.03)	.07
Other/unknown	0.78 (0.42-1.47)	.45
Housing status		
Other^a^	1 [Reference]	[Reference]
Shelter/street	0.73 (0.54-0.98)	.03
Clinical characteristics		
Charlson comorbidity index		
0-1	1 [Reference]	[Reference]
≥2	1.55 (1.10-2.18)	.001
Alcohol use disorder	1.23 (0.85-1.77)	.27
Other drug use disorder	0.92 (0.63-1.34)	.65
Serious mental illness^b^	0.86 (0.55-1.35)	.51
Medication prescriptions^c^		
Benzodiazepine	0.66 (0.20-2.14)	.49
Opioid^d^	0.64 (0.16-2.60)	.53
Antidepressant	0.49 (0.23-1.08)	.08
Antipsychotic	0.93 (0.32-2.66)	.89
Other sedating medications^e^	1.05 (0.32-3.47)	.94
Naloxone	1.19 (0.29-4.95)	.81
Addiction treatment–related characteristics		
Year of first OBAT encounter		
2008-2013	1 [Reference]	[Reference]
2014-2018	1.27 (0.90-1.79)	.17
Site of first OBAT encounter		
Clinic	1 [Reference]	[Reference]
Shelter/outreach	0.58 (0.36-0.93)	.03
OBAT attendance^c^		
Out of care	1 [Reference]	[Reference]
In care	0.34 (0.21-0.55)	<.001

Abbreviation: OBAT, office-based addiction treatment.

^a^ Includes assisted living facility, doubled up, housing with and without supportive services, motel, respite home, residential treatment center, skilled nursing facility, transitional housing, other housing status, and unknown housing status.

^b^ Includes a diagnosis of bipolar disorder and/or schizophrenia.

^c^ Time-varying, measured on a monthly basis.

^d^ Excludes buprenorphine.

^e^ Includes nonbenzodiazepine sedative-hypnotics and trazodone.

In the sensitivity analysis that included all 3 treatment-related covariates, past-month OBAT program attendance remained protective against all-cause mortality (aHR, 0.43; 95% CI, 0.24-0.78). In addition, past-month opioid abstinence (aHR, 0.37; 95% CI, 0.18-0.78) was independently associated with decreased hazard of all-cause mortality (eTable 3 in the Supplement). Results remained consistent in the sensitivity analysis using OBAT encounters as a continuous variable (eTable 4 in the Supplement).

### Addiction Treatment-related Outcomes

A total of 957 patients (65.2%) were initiated on buprenorphine therapy during the study period. The median time from the first OBAT encounter to buprenorphine initiation was 8 days (interquartile range [IQR], 0-137 days). Over a median of 4 years (IQR, 2.4-6.2 years) of follow-up, the median number of OBAT encounters was 4 (IQR, 0-17) (Table 1). Continuous OBAT retention was 45.2% at 1 month, 21.7% at 6 months, and 11.3% at 12 months (Figure). Hispanic ethnicity (adjusted OR [aOR], 1.61; 95% CI, 1.25-2.06) and other drug use disorders (aOR, 2.05; 95% CI, 1.54-2.77) were independently associated with increased OBAT treatment retention at 1 month. Literal homelessness (aOR, 0.79; 95% CI, 0.64-0.98), having serious mental illness (aOR, 0.71; 95% CI, 0.51-0.98), receiving other opioid prescriptions (aOR, 0.56; 95% CI, 0.31-0.98), and having an initial OBAT encounter in 2014 or later (aOR, 0.67; 95% CI, 0.53-0.86) were independently associated with decreased OBAT treatment retention at 1 month (Table 4).

**Figure.**
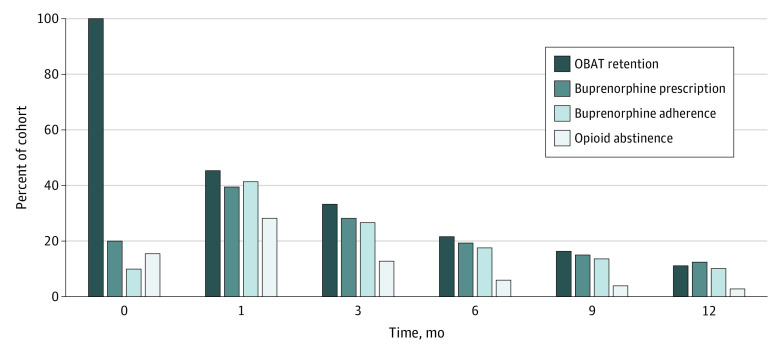
Continuous Addiction Treatment–Related Outcomes Over 12 Months Among People Experiencing Homelessness Who Engaged in an Office-Based Addiction Treatment (OBAT) Program Estimates of continuous OBAT program retention at the first OBAT encounter (time, 0) and 1, 3, 6, 9, and 12 months measured on a monthly basis.

**Table 4.** Baseline Characteristics Associated With One-Month Addiction Treatment Retention Among People Experiencing Homelessness Who Engaged in an OBAT Program^a^

Patient characteristic	Adjusted odds ratio (95% CI)	*P* value
Sociodemographic characteristics		
Age (per 10-y increment)	1.00 (0.99-1.01)	.48
Sex		
Female	1 [Reference]	[Reference]
Male	1.21 (0.95-1.54)	.12
Race/ethnicity		
White	1 [Reference]	[Reference]
Non-Hispanic Black	0.83 (0.59-1.17)	.29
Hispanic	1.56 (1.22-2.01)	<.001
Other/unknown	0.80 (0.52-1.22)	.30
Housing status		
Other^b^	1 [Reference]	[Reference]
Shelter/street	0.79 (0.64-0.98)	.04
Clinical characteristics		
Charlson comorbidity index		
0-1	1 [Reference]	[Reference]
≥2	0.91 (0.67-1.22)	.52
Alcohol use disorder	0.93 (0.70-1.23)	.61
Other drug use disorder	1.97 (1.47-2.66)	<.001
Serious mental illness^c^	0.71 (0.51-0.98)	.04
Medication prescriptions		
Benzodiazepine	1.41 (0.73-2.72)	.30
Opioid^d^	0.56 (0.31-0.98)	.05
Antidepressant	1.22 (0.90-1.67)	.21
Antipsychotic	0.94 (0.61-1.44)	.78
Other sedating medications^e^	1.45 (0.85-2.48)	.17
Naloxone	1.11 (0.67-1.83)	.67
Addiction treatment–related characteristics		
Year of first OBAT encounter		
2008-2013	1 [Reference]	[Reference]
2014-2018	0.67 (0.53-0.86)	<.001
Site of first OBAT encounter		
Clinic	1 [Reference]	[Reference]
Shelter/outreach	1.30 (0.99-1.72)	.06

Abbreviation: OBAT, office-based addiction treatment.

^a^ A total of 666 individuals were retained in the OBAT program at 1 month.

^b^ Includes assisted living facility, doubled up, housing with and without supportive services, motel, respite home, residential treatment center, skilled nursing facility, transitional housing, other housing status, and unknown housing status.

^c^ Includes a diagnosis of bipolar disorder and/or schizophrenia.

^d^ Excludes buprenorphine.

^e^ Includes nonbenzodiazepine sedative-hypnotics and trazodone.

Continuous buprenorphine adherence was 41.5% at 1 month, 17.6% at 6 months, and 10.2% at 12 months (Figure). The median number and percentage of positive buprenorphine toxicologic tests per person were 4 (IQR, 1-17) and 100% (IQR 80%-100%) (Table 1). Continuous opioid abstinence was 28.3% at 1 month, 6.1% at 6 months, and 2.9% at 12 months (Figure). The median number and percentage of negative opioid toxicologic tests were 2 (IQR, 0-9.5) and 61% (IQR, 20%-100%) (Table 1). Point-prevalent OBAT retention, buprenorphine adherence, and opioid abstinence over 12 months are shown in eFigure 2 in the Supplement.

## Discussion

This large cohort study of people experiencing homelessness who engaged in a tailored OBAT program revealed 3 major findings. First, the overall mortality rate was high, with a substantial number of patients dying from drug overdose. Second, OBAT program attendance was associated with decreased mortality despite complete opioid abstinence being uncommon. Third, continuous retention in addiction care was low, with considerable loss to follow-up within the first month of care.

The all-cause mortality in this cohort was 12-fold higher than in a similarly aged general population^24^ and 2-fold higher than in a similarly aged homeless population,^1^ although it was only marginally higher than another OBAT program in Boston over the same time period.^25^ Drug overdose was the leading cause of death, accounting for approximately half of the deaths for which the underlying cause was available. Opioids contributed to all of these overdose deaths, illustrating the relapsing nature of opioid addiction.

Past-month OBAT program attendance was independently associated with reduced mortality despite the fact that few patients remained abstinent from illicit opioids. This measure of monthly OBAT attendance did not necessitate continuous retention in care, which is difficult to achieve for individuals with OUD, particularly those experiencing homelessness.^12^ A sensitivity analysis demonstrated that program attendance remained protective independent of buprenorphine adherence and opioid abstinence. These findings support a low-threshold harm reduction approach that prioritizes engagement in addiction care whenever possible regardless of lapses in previous attendance or ongoing illicit drug use.^26,27,28^

Patients who were living in a shelter or on the street were less likely than housed individuals to be retained in OBAT care at 1 month but also less likely to die during follow-up. This finding could relate to the circumstances in which substance use occurs, with those in shelters or on the street having people nearby to respond to an overdose, whereas those in housing may be using drugs alone. Taken together, these findings suggest the need for efforts to enhance treatment retention among unhoused individuals while raising awareness about the potentially heightened risk of fatal overdose in isolated residential settings.

Although OBAT retention in this study was lower than that observed in more traditional office-based settings where the percentage of homeless patients was substantially lower,^29,30,31,32^ the retention was similar to that in a previous study evaluating addiction treatment retention in the homeless population.^12^ Continuous care retention may be an unrealistic expectation for people experiencing homelessness whose ability to consistently attend appointments may be disproportionately affected by incarceration, competing priorities, and/or return to drug use. Despite these low retention rates, there was a subset of patients who were continuously retained and more who were intermittently retained who may not have been able to access such care—and its associated survival benefit—elsewhere.

As has been reported in previous studies evaluating buprenorphine treatment retention,^31,32^ the largest dropoff in addiction care was within the first month after study entry, with less than half of all patients returning for follow-up. This finding suggests that interventions aimed toward retaining patients at high risk of dropping out of care should be introduced during the first OBAT encounter. Baseline characteristics associated with increased 1-month treatment retention included Hispanic ethnicity and the presence of other drug use disorders. Hispanic ethnicity may be associated with increased retention owing to community partnerships and the considerable number of bicultural and bilingual OBAT staff at BHCHP. Baseline characteristics associated with reduced 1-month retention included literal homelessness, serious mental illness, and an initial OBAT encounter after 2013. Patients who had their first OBAT encounter after 2013 may have been less likely to follow-up owing to several related reasons. Fentanyl was introduced into the drug supply during this period, overdoses increased, and BHCHP responded by lowering the threshold for OBAT program entry. Future research should investigate the outcomes of interventions to improve retention in addiction care within this population, such as ensuring that intakes do not delay buprenorphine prescribing, incorporating peer support,^33^ using telehealth for OBAT engagement,^34^ and proactive outreach for individuals who drop out of care.^35^

### Limitations

This study has limitations. This study evaluated adults who received care in BHCHP’s OBAT program, so the findings may not be generalizable to homeless individuals who avoid care or to homeless adults in other cities. In addition, the observational nature of the study introduces the possibility of confounding by unmeasured variables, such as the severity of addiction, periods of incarceration, and social supports.

Although supplemental analyses suggested that opioid abstinence had protective associations with mortality, these findings should be interpreted with caution given the difficulty of disentangling the combined effects of OBAT follow-up, buprenorphine adherence, and opioid abstinence. To help guide clinical care and reduce mortality risk in this vulnerable population, future studies should attempt to examine the comparative importance of each on mortality.

Other limitations include those related to using electronic health record data, such as potential coding errors, validation of variable measurement, and the inability to capture data on patients if they transfer care to other health care systems.

## Conclusions

In this large cohort of treatment-seeking homeless individuals with OUD, mortality rates were high, with a substantial burden of deaths caused by drug overdose. OBAT program retention was low, particularly within the first month of follow-up, but program attendance was associated with reduced mortality irrespective of buprenorphine adherence and opioid abstinence. We suggest that OBAT programs for similarly high-risk populations recognize the challenges with and importance of program attendance. Interventions aimed toward promoting increased OBAT program attendance among people experiencing homelessness are needed.

## Notes

Supplement. **eTable 1.** *International Classification of Diseases–10th Edition* (*ICD-10*) Codes Used to Define Cause of Death **eTable 2.** List of *ICD* Code Algorithms and Their Sources **eFigure 1.** Mortality Rates Among People Experiencing Homelessness Who Engaged in an Office-Based Addiction Treatment Program **eTable 3.** Sensitivity Analysis Including OBAT Attendance, Buprenorphine Adherence, and Opioid Abstinence **eTable 4.** Sensitivity Analysis Including OBAT Attendance as a Continuous Variable **eFigure 2.** Point-Prevalent Addiction Treatment-Related Outcomes Over 12 Months Among People Experiencing Homelessness Who Engaged in an Office-Based Addiction Treatment (OBAT) Program **eReferences.**
