# Error in Abstract and Methods

**DOI:** 10.1001/jamanetworkopen.2021.28112

**Published:** 2021-08-27

In the Original Investigation titled “Office-Based Addiction Treatment Retention and Mortality Among People Experiencing Homelessness,”^1^ published March 4, 2021, there were errors in the date ranges in the Abstract and Methods. The dates for the cohort of people with 1 or more office-based addiction treatment encounters given as “January 1” and “December 21, 2018,” should have been “January 1, 2008,” and December 31, 2018.” This article has been corrected.^1^
