# Error in Table 2

**DOI:** 10.1001/jamanetworkopen.2023.45320

**Published:** 2023-11-09

In the Original Investigation titled “Office-Based Addiction Treatment Retention and Mortality Among People Experiencing Homelessness,”^1^ published March 4, 2021, there was an error in Table 2. The number and percentage in “Drug overdose” for Alcohol given as “32 (36.8)” should have been “15 (17.2),” and the number and percentage for Cocaine given as “15 (7.2)” should have been “32 (36.8).” In addition, the rows were rearranged to maintain descending numeric order. This article has been corrected.^1^ This article was previously corrected to fix an error in the Abstract and Methods.^2^
