# Supplementary material for: Office-Based Addiction Treatment Retention and Mortality Among People Experiencing Homelessness
Source: JAMA Netw Open. 2021 Mar 4;4(3):e210477. doi: 10.1001/jamanetworkopen.2021.0477 (PMC7933994; doi:10.1001/jamanetworkopen.2021.0477)
Supplement: Supplement. — eTable 1. International Classification of Diseases–10th Edition (ICD-10) Codes Used to Define Cause of Death eTable 2. List of ICD Code Algorithms and Their Sources eFigure 1. Mortality Rates Among People Experiencing Homelessness Who Engaged in an Office-Based Addiction Treatment Program eTable 3. Sensitivity Analysis Including OBAT Attendance, Buprenorphine Adherence, and Opioid Abstinence eTable 4. Sensitivity Analysis Including OBAT Attendance as a Continuous Variable eFigure 2. Point-Prevalent Addiction Treatment-Related Outcomes Over 12 Months Among People Experiencing Homelessness Who Engaged in an Office-Based Addiction Treatment (OBAT) Program eReferences. [file jamanetwopen-e210477-s001.pdf]

Supplementary Online Content

Fine DR, Lewis E, Weinstock K, Wright J, Gaeta JM, Baggett TP. Office-based addiction treatment retention and mortality among people experiencing homelessness. *JAMA Netw Open*. 2021;4(3):e210477. doi:10.1001/jamanetworkopen.2021.0477

eTable 1. *International Classification of Diseases–10th Edition* (*ICD-10*) Codes Used to Define Cause of Death

eTable 2. List of *ICD* Code Algorithms and Their Sources

eFigure 1. Mortality Rates Among People Experiencing Homelessness Who Engaged in an Office-Based Addiction Treatment Program

eTable 3. Sensitivity Analysis Including OBAT Attendance, Buprenorphine Adherence, and Opioid Abstinence

eTable 4. Sensitivity Analysis Including OBAT Attendance as a Continuous Variable

eFigure 2. Point-Prevalent Addiction Treatment-Related Outcomes Over 12 Months Among People Experiencing Homelessness Who Engaged in an Office-Based Addiction Treatment (OBAT) Program

eReferences

This supplementary material has been provided by the authors to give readers additional information about their work.

© 2021 Fine DR et al. *JAMA Network Open.*

eTable 1. ***International Classification of Diseases* – *10th Edition (ICD-10)* codes used to define cause of death**

Underlying cause of death	ICD-10 codes
External causes	V01-Y98
Drug overdose	X40-X44, Y10-Y14
Alcohol poisoning	X45, Y15
Suicide	X60-X84, Y87.0
Homicide	X85-Y09, Y87.1
Accidents (unintentional injuries)	V01-V99, Y85
Other accidents (non-poisoning)	W00-X39, X50-X59, Y86
Natural causes	
Sepsis	A40-A41
Viral hepatitis	B15-B19
HIV disease	B20-B24
Cancer	C00-C97
Diabetes	E10-E14
Psychoactive substance use disorder	F10-F19
Anoxic brain injury	G93.1
Other diseases of the nervous system	G12, G93
Heart disease	I00-I09, I11, I13, I20-I51
Cerebrovascular disease	I60-I69
Influenza and pneumonia	J10-J18
Chronic lower respiratory diseases	J40-J47
Other diseases of the respiratory system	J81
Liver disease	K70-K77
Nephritis, nephrotic syndrome, and nephrosis	N00-N07, N17-N19, N25-N27
Congenital malformations of the heart	Q23
Ill-defined conditions	R00-R99, K229

Source: Centers for Disease Control and Prevention. ICD-10 Cause-of-Death Lists for Tabulating Mortality Statistics. National Center for Health Statistics. 2005. https://www.in.gov/isdh/reports/mortality/2005/im9_2002_pdf.pdf

© 2021 Fine DR et al. *JAMA Network Open.*

eTable 2. List of *ICD* code algorithms and their sources		
**Covariate**	**ICD Code Algorithm**	**Source**
Alcohol use disorder	**ICD 9 codes:** 291.x. 303.0x, 303.9x, 305.x, 357.5, 425.5, 535.30, 535.31, 571.1, 571.2, 571.3<br>**ICD 10 codes:** E860.0, F10.x	Heslin KC et al.[1]
Cardiovascular disease	**ICD-9 codes:** 410.x, 412.x,<br>**ICD-10 codes:** I21.x, I22.x, I25.2	Quan et al.[2]
Cerebrovascular disease	**ICD-9 codes:** 362.34, 430.x–438.x<br>**ICD-10 codes:** G45.x, G46.x, H34.0, I60.x–I69.x	Quan et al.[2]
Chronic Kidney disease	**ICD-9 codes:** 403.01, 403.11, 403.91, 404.02, 404.03, 404.12, 404.13, 404.92, 404.93, 582.x, 583.0–583.7, 585.x, 586.x, 588.0, V42.0, V45.1, V56.x<br>**ICD-10 codes:** I12.0, I13.1, N03.2–N03.7, N05.2– N05.7, N18.x, N19.x, N25.0, Z49.0– Z49.2, Z94.0, Z99.2	Quan et al.[2]
Chronic lung disease	**ICD-9 codes:** 416.8, 416.9, 490.x–505.x, 506.4, 508.1, 508.8<br>**ICD-10 codes:** I27.8, I27.9, J40.x–J47.x, J60.x–J67.x, J68.4, J70.1, J70.3	Quan et al.[2]
Cold related injury		--
Frostbite	**ICD-9 codes:** 991.0, 991.1, 991.2, 991.3<br>**ICD-10 codes:** T33, T34	
Hypothermia	**ICD-9 codes:** 991.6<br>**ICD-10 codes:** T68	
Immersion Foot	**ICD-9 codes:** 991.4<br>**ICD-10 codes:** T69.02	
Congestive heart failure	**ICD-9 codes:** 398.91, 402.01, 402.11, 402.91, 404.01, 404.03, 404.11, 404.13, 404.91, 404.93, 425.4–425.9, 428.x<br>**ICD-10 codes:** I09.9, I11.0, I13.0, I13.2, I25.5, I42.0, I42.5–I42.9, I43.x, I50.x, P29.0	Quan et al.[2]
Hepatitis C infection	**ICD-9 codes:** 070.41, 070.44, 070.51, 070.54, 070.7x<br>**ICD-10 codes:** B17.1, B18.2, B19.2	Niu B et al.[3]
HIV	**ICD-9 codes:** 042.x–044.x, V08<br>**ICD10 codes:** B20.x–B22.x, B24.x, Z21	Quan et al.[2]
Liver disease	**ICD-9 codes:** 070.22, 070.23, 070.32, 070.33, 070.6, 070.9, 456.0–456.2, 570.x, 571.x, 572.2–572.8, 573.3, 573.4, 573.8, 573.9, V42.7<br>**ICD-10 Codes:** B18.0, B18.1, I85.x, I86.4, I98.2, K70.x, K71.1, K71.3– K71.5, K71.7, K72.x– K74.x, K76.0, K76.2–K76.9, Z94.4	Adapted from Quan et al.[2] (excluded hepatitis C codes)
Malignancy	**ICD-9 codes:** 140.x–172.x, 174.x–195.8, 200.x–208.x, 238.6, 196.x–199.x<br>**ICD-10 codes:** C00.x–C26.x, C30.x–C34.x, C37.x– C41.x, C43.x, C45.x–C58.x, C60.x–C76.x, C81.x–C85.x, C88.x, C90.x–C97.x, C77.x–C80.x	Quan et al.[2]

© 2021 Fine DR et al. *JAMA Network Open.*

Covariate	ICD Code Algorithm	Source
Other Drug Use Disorders		Heslin KC et al.[1]
Amphetamine use disorder	**ICD-9 codes:** 304.4x, 305.7x<br>**ICD-10 codes:** F15.x	
Cannabis use disorder	**ICD-9 codes:** 304.3x, 305.2x<br>**ICD-10 codes:** F12.x	
Cocaine use disorder	**ICD-9 codes:** 304.2, 305.6, 968.5<br>**ICD-10 codes:** E938.5, F14.x	
Hallucinogen use disorder	**ICD-9 codes:** 304.5x, 305.3x, 969.6<br>**ICD-10 codes:** E939.6, F16.x	
Sedative use disorder	**ICD-9 codes:** 304.1x, 305.4x<br>**ICD-10 codes:** F13.x	
Other/polysubstance use disorder	**ICD-9 codes:** 304.6x, 304,8x, 304.9x, 305.9x, 648.3x, V654.2<br>**ICD-10 codes:** F18.x, F19.x	
Serious bacterial infection		Ronin, MV and Herzig, SJ[4]; Schranz et al.[5]
Endocarditis	**ICD-9 codes:** 036.42, 098.84, 112.81, 115.04, 115.14, 115.94, 421.0, 421.1, 421.9, 424.90, 424.91, 424.99<br>**ICD-10 codes:** A39.51, A54.83, B37.6, I33.0, I33.9, I38, I39	
Epidural Abscess	**ICD-9 codes:** 324.0, 324.1, 324.9<br>**ICD-10 codes:** G06, G07, G08	
Septic Arthritis	**ICD-9 codes:** 003.23, 036.82, 711.0x-711.4x, 711.6x, 711.9x<br>**ICD-10 codes:** A02.23, A39.83, M00.0x, M00.1x, M00.2x, M00.8x, M00.9, M01.Xx	
Osteomyelitis	**ICD-9 codes:** 003.24, 730.0x, 730.2x, 730.8x, 730.9x<br>**ICD-10 codes:** A02.24, M86.0x, M86.1x, M86.2x, M86.8, M86.8X, M86.9	
Serious Mental Illness		--
Bipolar disorder	**ICD-9 codes:** 296.0x, 296.4x, 296.5x, 296.6x, 296.7, 296.8x<br>**ICD-10 codes:** F31	
Schizoprenia	**ICD-9 codes:** 295.xx<br>**ICD-10 codes:** F20, F25	

© 2021 Fine DR et al. *JAMA Network Open.*

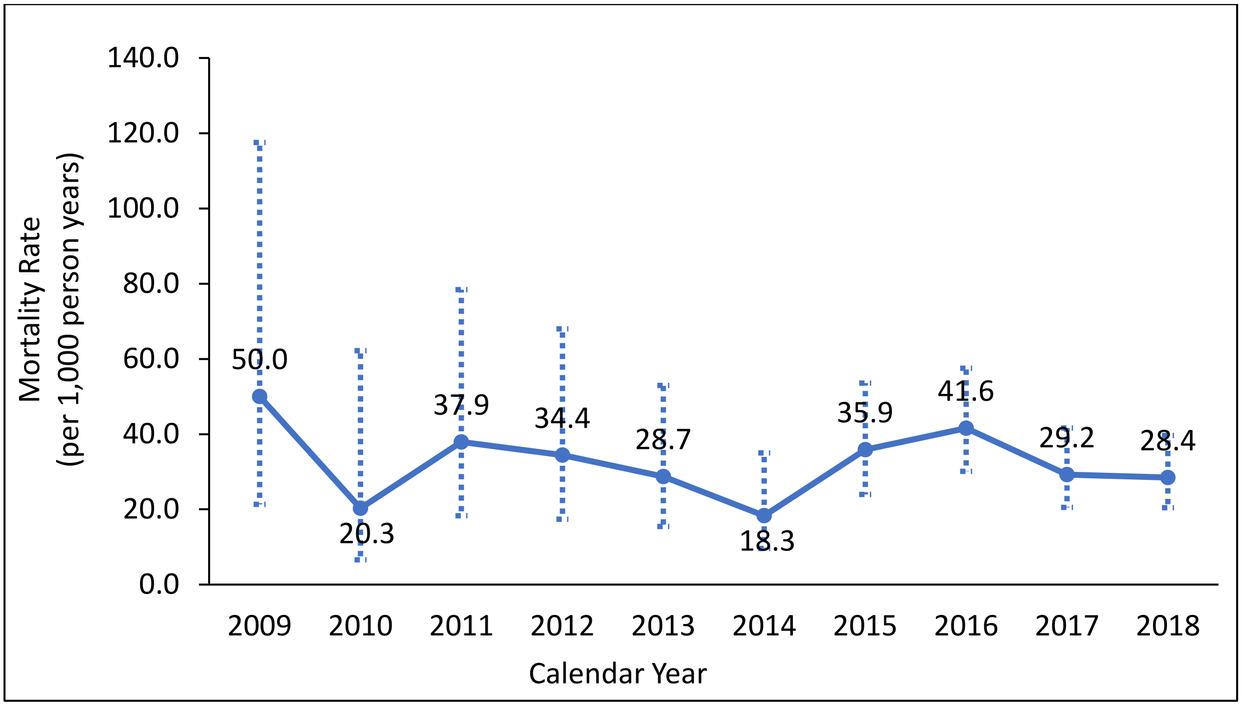

eFigure 1. Mortality rates among people experiencing homelessness who engaged in an office-based addiction treatment program. Point estimates represent deaths per 1,000 person-years and vertical blue dashed-lines represent 95% confidence intervals.

© 2021 Fine DR et al. *JAMA Network Open.*

eTable 3. Sensitivity analysis including OBAT attendance, buprenorphine adherence, and opioid abstinence		
**Addiction treatment-related characteristics**	**Adjusted[a] Hazard Ratio (95% CI)**	**p value**
OBAT attendance		
Out of care	ref	ref
In care	0.43 (0.24, 0.78)	<0.01
Buprenorphine toxicology[b]		
Buprenorphine negative	ref	ref
Buprenorphine positive	0.47 (0.18, 1.21)	0.12
Opioid toxicology[b]		
Opioid positive	ref	ref
Opioid negative	0.37 (0.18, 0.78)	<0.01

[a] Model also adjusted for age, sex, race/ethnicity, housing status, Charlson comorbidity index, alcohol use disorder, other drug use disorder, serious mental illness, year of first OBAT encounter, and site of first OBAT encounter
[b] Missing indicator method used for buprenorphine toxicology and opioid toxicology results

© 2021 Fine DR et al. *JAMA Network Open.*

**eTable 4. Sensitivity analysis including OBAT attendance as a continuous variable**		
**Addiction treatment-related characteristic**	**Adjusted[a] Hazard Ratio (95% CI)**	**p value**
OBAT attendance (per additional visit)	0.96 (0.95, 0.98)	<0.01
[a] Model also adjusted for age, sex, race/ethnicity, housing status, Charlson comorbidity index, alcohol use disorder, other drug use disorder, serious mental illness, year of first OBAT encounter, and site of first OBAT encounter		

© 2021 Fine DR et al. *JAMA Network Open.*

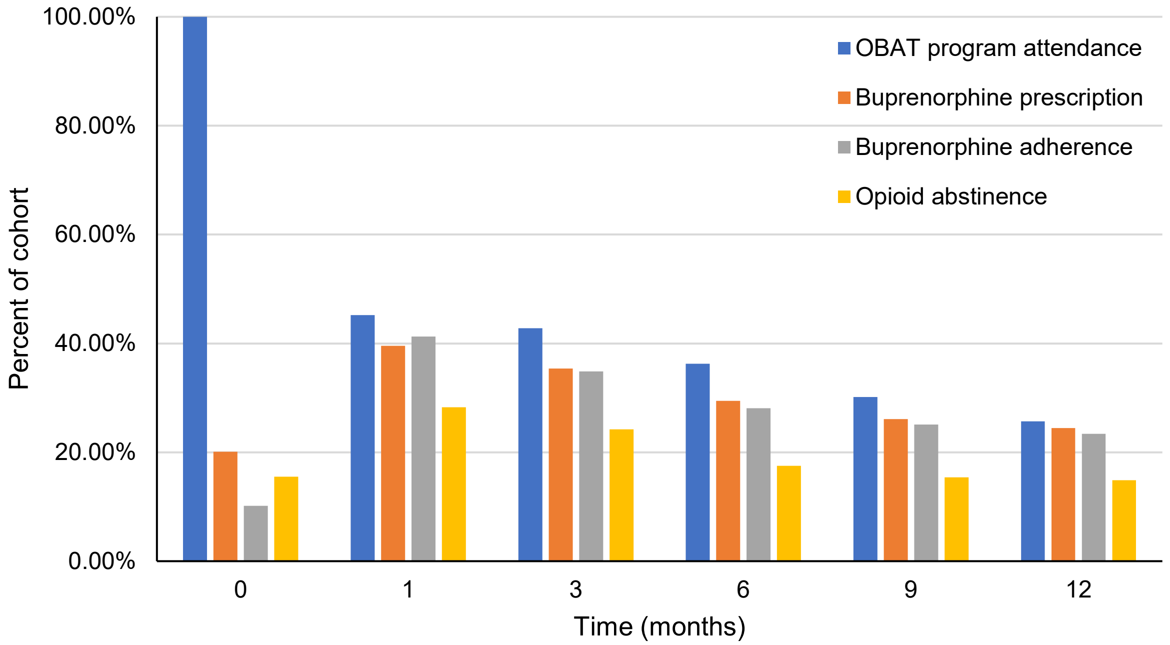

eFigure 2. Point-prevalent addiction treatment-related outcomes over 12 months among people experiencing homelessness who engaged in an office-based addiction treatment (OBAT) program. Estimates of point-prevalent OBAT program attendance (blue bars), point-prevalent buprenorphine prescriptions (orange bars), point-prevalent positive buprenorphine toxicology results (grey bars), and point-prevalent negative opioid toxicology results (yellow bars) at the first OBAT encounter (Time=0) and 1, 3, 6, 9, and 12 months.

© 2021 Fine DR et al. *JAMA Network Open.*

eReferences

1. Heslin KC EA, Steiner CA. Hospitalizations Involving Mental and Substance Use Disorders Among Adults, 2012. HCUP Statistical Brief #191. *Agency for Healthcare Research and Quality.* 2015.
2. Quan H, Sundararajan V, Halfon P, et al. Coding algorithms for defining comorbidities in ICD-9-CM and ICD-10 administrative data. *Med Care.* 2005;43(11):1130-1139.
3. Niu B, Forde KA, Goldberg DS. Coding algorithms for identifying patients with cirrhosis and hepatitis B or C virus using administrative data. *Pharmacoepidemiol Drug Saf.* 2015;24(1):107-111.
4. Ronan MV, Herzig SJ. Hospitalizations Related To Opioid Abuse/Dependence And Associated Serious Infections Increased Sharply, 2002-12. *Health Aff (Millwood).* 2016;35(5):832-837.
5. Schranz AJ, Fleischauer A, Chu VH, Wu LT, Rosen DL. Trends in Drug Use-Associated Infective Endocarditis and Heart Valve Surgery, 2007 to 2017: A Study of Statewide Discharge Data. *Ann Intern Med.* 2018.

© 2021 Fine DR et al. *JAMA Network Open.*
